# 

**Published:** 1906-03

**Authors:** 


### AMERICAN INTERNATIONAL CONGRESS ON TUBERCULOSIS, 1906.

The American International Congress on Tuberculosis is hereby called to meet in New York City November 14, 1906. A joint session of the Congress and the Medico-Legal Society will be held. Titles of papers and the names of members who will attend should be sent to the Secretary at as early date as possible. Full particulars will be published in the medical and lay press next month.
By order of the EXECUTIVE COMMITTEE.

Approved:

F. E. DANIEL, M. D., President, Austin, Texas.

M. M. SMITH, General Secretary, Austin, Texas.

[Exchanges and other publications will please copy this announcement.—EDITOR.]

